# Association of rituximab with graphene oxide confers direct cytotoxicity for CD20-positive lymphoma cells

**DOI:** 10.18632/oncotarget.7230

**Published:** 2016-02-07

**Authors:** Chengke Luo, Zhenghao Deng, Lan Li, Frederic Clayton, Alexander L. Chen, Ran Wei, Rodney Miles, Deborah M. Stephens, Martha Glenn, Xiyang Wang, Peter E. Jensen, Xinjian Chen

**Affiliations:** ^1^ Department of Spine Surgery, Xiangya Hospital of Central-South University, Changsha, China; ^2^ Department of Pathology, University of Utah, Salt Lake City, Utah, USA; ^3^ Huntsman Cancer Institute, Hematology Division, Department of Internal Medicine, University of Utah, Salt Lake City, Utah, USA

**Keywords:** Rituximab, CD20, graphene oxide, non-Hodgkin lymphoma

## Abstract

Non-Hodgkin lymphoma (NHL) is one of the most common hematologic malignancies among adults for which the chimeric monoclonal anti-CD20 antibody (Ab) rituximab (RTX) is used as first-line therapy. As RTX itself is not directly cytotoxic but relies on host immune effector mechanisms or chemotherapeutic agents to attack target cells, its therapeutic capacity may become limited when host effector mechanisms are compromised. Currently, refractory disease and relapse with NHL are still common, highlighting the need for novel anti-CD20 antibody strategies with superior therapeutic efficacy over current protocols. We hypothesized that making RTX directly cytotoxic might improve the therapeutic efficacy. Graphene oxide (GO) has recently emerged as a highly attractive nanomaterial for biomedical applications; and several studies have reported cytotoxic effect of GO on benign and malignant cells *in vitro*. Herein, we report that RTX can be stably associated with GO, and that GO-associated RTX (RTX/GO) demonstrates remarkably high avidity for CD20. Binding of GO-associated RTX to CD20-positive lymphoma cells induces CD20 capping and target cell death through an actin dependent mechanism. *In vivo,* GO-associated RTX, but not free RTX, quickly eliminates high-grade lymphomas in the absence of host effector mechanisms in a xenograft lymphoma mouse model. Our findings represent the first demonstration of using GO-associated antibody as effective cytotoxic therapy for human B cell malignancies in the absence of chemotherapy, and these findings could have important clinical implications.

## INTRODUCTION

Non-Hodgkin lymphoma (NHL) is one of the most common hematologic malignancies among adults, for which the chimeric monoclonal anti-CD20 antibody (Ab) rituximab (RTX) is used as first-line therapy [1]. While the therapeutic mechanism of RTX is a subject of active investigation, there has been convincing evidence showing that RTX does not promote direct killing of target cells, but instead depends on participation of the host immune effector mechanisms such as Ab-dependent cell-mediated cytotoxicity (ADCC), complement-dependent cytotoxicity (CDC), or phagocytosis to attack target cells. [2-4]. The lack of direct cytotoxicity may limit therapeutic efficacy of RTX when host effector functions are ineffective, such as in patients whose FcγRs have low affinity for IgG due to genetic polymorphisms [5]. In addition, in patients with large tumor burdens, host effector mechanisms supporting Ab-mediated tumor elimination can be exhausted, resulting in binding of RTX to CD20 without subsequent elimination of the target cells [6, 7]. RTX-bound CD20 can be readily stripped or shaved from the cell surface by macrophages, resulting in downregulation of CD20 expression, further compromising RTX therapeutic efficacy [8, 9]. The overall response rate of NHL patients to RTX monotherapy is very low [10, 11], suggesting limited efficacy of the host effector mechanisms in eliminating lymphoma cells. Combining chemotherapy with RTX increases response rates, but chemotherapy is often associated with serious side effects along with damage to leukocytes, including lymphocytes, which could hamper antitumor immunity [12]. Currently, refractory disease and relapse with NHL are still common [13, 14], highlighting the need for novel anti-CD20 Ab strategies with superior therapeutic capacity over current RTX protocols [4, 15]. We hypothesized that making RTX cytotoxic with the capacity to directly eliminate lymphoma cells would improve therapeutic efficacy of the antibody. In light of previous reports showing that crosslinking CD20 (CD20XL) with RTX and a second antibody kills malignant B cells *in vitro* [16, 17], we sought to make RTX cytotoxic by increasing antibody valence through associating the antibody to a nanomaterial, graphene oxide (GO).

GO has recently attracted intense interest of research owing to its unique physical, chemical and biological properties, as well as the potential for biomedical applications [18, 19]. GO has a two-dimensional single-atom-thick nanosheet structure composed of a monomolecular layer of aromatic carbon rings with oxygen containing moieties. Because of its small size with relatively large surface area, GO and its derivatives can be loaded with drugs, nucleic acids, or contrast dyes for drug or gene delivery, cellular imaging or photothermal ablation of tumors [20-23], In addition to vehicle function, studies have also reported cytotoxic effects of GO *in vitro* on both benign and malignant human cells, including cancer stem cells [24-26]. GO can cause cytotoxicity by oxidative stress and mitochondrial activation [27]. GO can also induce rupture of liposomes and disrupt the integrity of bacterial cell membranes [28, 29]. GO-induced cytotoxicity appears to be dose-dependent: at low concentrations, GO has no significant cytotoxicity but causes oxidative stress and induces a loss of cell viability at high concentrations [24]. GO has not been studied as a scaffold material for formation of multivalent antibodies. Given the molecular features of GO, there is a possibility that antibody molecules may be able to stably associate with GO through non-covalent interactions such as ionic and hydrogen bonds, and hydrophobic interactions. The side chains of aromatic amino acids such as phenylalanine, tyrosine, and tryptophan contained in antibody molecules may provide surfaces of electrostatic potential to interact with the aromatic rings of GO through *π* -*π* stacking [30]. If RTX can stably associate with GO to form multivalent antibodies, GO-associated RTX may have the capacity to crosslink CD20 and kill CD20-positive target cells as suggested in previous studies [16, 31, 32]. In addition, targeted delivery of GO to CD20-positive target cells may allow GO to achieve local high concentrations to kill the target cells by oxidative stress associated cell membrane damage. In the current report, we studied the non-covalent association between RTX and GO, examined the reactivity of GO-associated RTX (RTX/GO), and established the capacity of RTX/GO to eliminate CD20^+^ lymphomas.

## RESULTS

### Rituximab can be stably loaded onto graphene oxide

**Figure 1:**
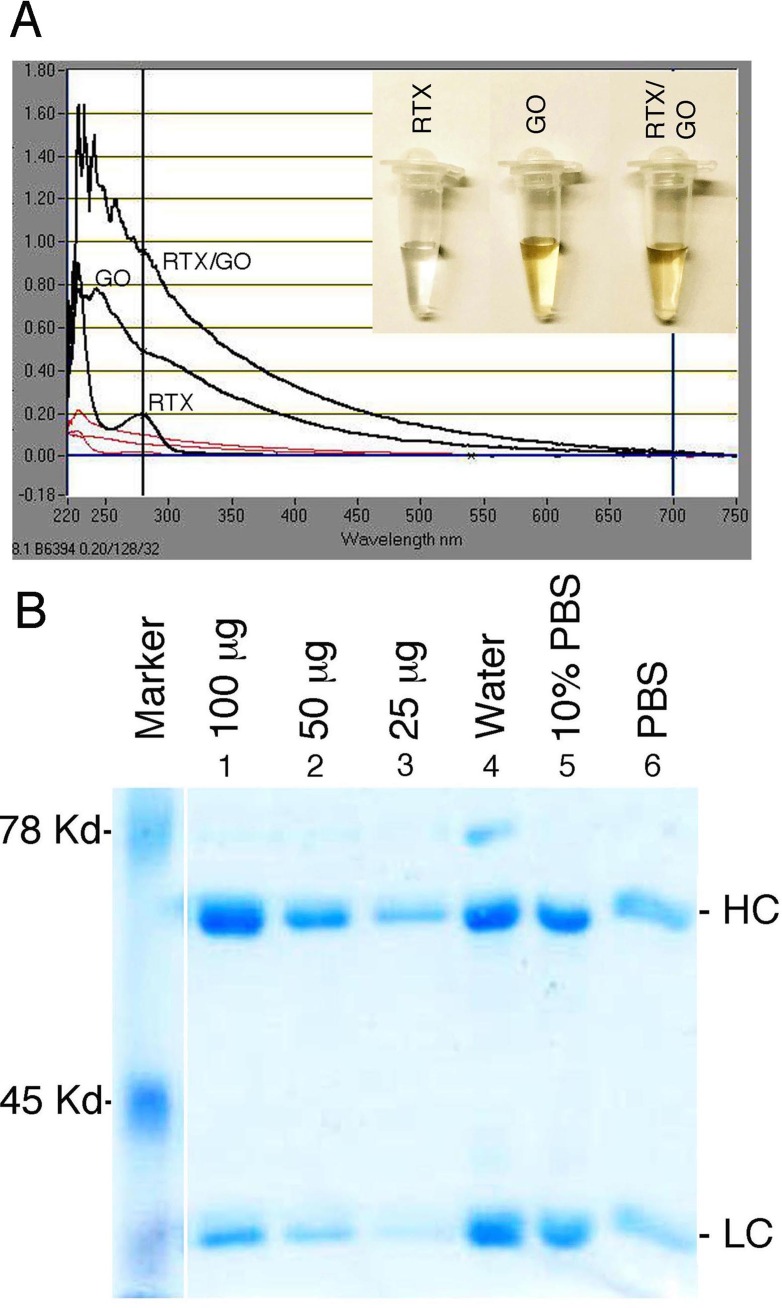
Association RTX with GO: The UV-Vis spectrum absorbance of free RTX, GO, and RTX/GO mixture is overlaid **A.** Indicated quantity of RTX was loaded in lanes 1 through 3. RTX eluted from RTX/GO mixture that had been incubated at 37°C overnight in water, 10% and undiluted PBS were loaded in lanes 4, 5 and 6 respectively. HC and LC indicate the heavy and light chain of RTX respectively **B.**

Consisting of sp2-hybridized carbon rings with hydroxyl and carboxyl groups, GO has the potential to noncovalently interact with antibody molecules through π-stacking, hydrophobic interactions, as well as with hydrogen and ionic bonds [18, 21]. To determine whether RTX and GO can stably associate with each other through noncovalent bonds, vigorously sonicated and 0.22μ-filtered GO (Figure 1A, insert) was mixed with RTX in water, 10% PBS, or undiluted PBS, and incubated at 37°C overnight under constant agitation. On UV-Vis spectroscopy, free RTX absorption peaked at 280 nm, whereas free GO had a broad absorption spectrum that peaked at 230 nm as previously reported [33]. The mixture of RTX and GO (RTX/GO) gave rise to an absorption spectrum similar to that of free GO but with substantially increased magnitude (Figure 1A), suggesting an association between RTX and GO. To quantitate the stoichiometric association between GO and RTX, RTX/GO mixtures were centrifuged and thoroughly washed with PBS in 37°C to obtain RTX-associated GO. The RTX was eluted from RTX-GO complexes with denaturing buffer and examined by SDS PAGE. As compared with the known concentrations of RTX loaded in parallel electrophoresis lanes, approximately 100 μg of RTX was found to be associated with 20 μg of GO when the mixture was prepared in water or 10% PBS (Figure 1B), giving rise to a 5:1 mass ratio of RTX:GO. Preparing the mixture in undiluted PBS, however, substantially reduced the amount of RTX associated with GO (Figure 1B). These results demonstrate that low salt conditions promote the formation of stable complexes between RTX and GO which do not dissociate in buffered normal saline solutions at body temperature (37°C).

### GO-associated RTX binds to CD20 with high avidity and induces CD20 capping

**Figure 2:**
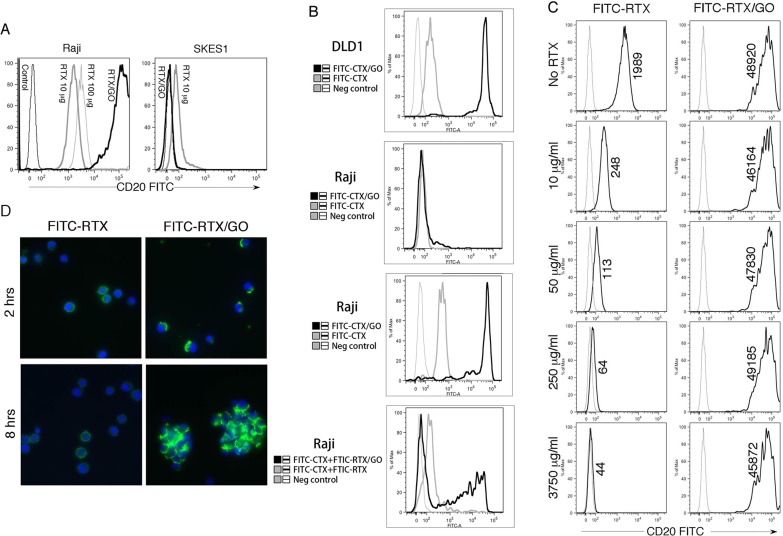
CD20 Reactivity of RTX/GO: Raji cells and a CD20^neg^ Ewing sarcoma cell line SKES1 were stained with FITC-RTX at 10 μg/ml or 100 μg/ml, or RTX/GO (10 μg/2 μg/ml) mixture The cells were analyzed by flow cytometry **A.** Human colon carcinoma cell line DLD1 cells and Raji cells were stained with the indicated treatment and analyzed by flow cytometry **B.** Raji cells were incubated for 30′ with unlabeled RTX at the indicated concentrations before being stained with free FITC-RTX or FITC-RTX/GO for 30′. The numbers represent the geometric mean of fluorescence intensity as detected by flow cytometry **C.** Raji cells were stained with free FITC-RTX or FITC-RTX/GO mixture for 2 hours or 8 hours and examined by confocal microscopy with a 10x objet lens **D.** The experiments were repeated at least three times.

Since RTX and GO form stable, non-covalent associations through a stochastic process, RTX reactivity with CD20 might be affected by interference with the antigen-binding site of RTX. To test this possibility, RTX was conjugated with FITC and used to generate FITC-RTX/GO complexes. This FITC-RTX/GO mixture was used to stain a CD20^+^ Burkitt lymphoma cell line, Raji cells, and FITC-RTX was used as a control. Both free RTX and RTX/GO positively stained Raji cells, but the staining derived from FITC-RTX/GO was much (50-100 fold) brighter (Figure 2A). The difference in staining intensity was not due to differences in the overall concentration of RTX, as increasing the concentration of free RTX by ten fold only slightly increased the intensity of staining. RTX/GO staining was CD20 specific because RTX/GO did not stain a CD20^neg^ Ewing sarcoma cell line, SKES1 (Figure 2A). These results indicate that stochastic binding of RTX to GO does not abolish RTX reactivity to CD20, but rather substantially increases the binding capacity of RTX to CD20 expressing cells. To rule out the possibility that only a small fraction of FITC-RTX/GO actually chelates CD20, and that the strong fluorescent signals result from binding of the highly fluorescence particles to the cells, we used a dilution assay. GO was incubated with a combination of FITC-conjugated cetuximab (Erbitux, CTX, which is specific for EGFR) and FITC-conjugated RTX at a 1:1 ratio to generate FITC-RTX+FITC-CTX/GO. FITC-CTX/GO stained the EGFR-positive human colon carcinoma cell line DLD1 cells brightly (Figure 2B) but only weakly stained a small fraction of Raji cells. While FITC-RTX/GO stained Raji cells strongly, FITC-RTX+FITC-CTX/GO gave rise to weaker staining, despite similar particle fluorescence. The fraction of fluorescent cells was reduced by ∼50% and the positive cells had lower mean fluorescence intensity (Figure 2B). This result suggests that reduced particle valence of anti-CD20 impairs CD20-mediated binding of RTX-loaded GO nanoparticles to Raji cells.

The enhancement in CD20 binding suggests an increase in CD20 avidity of RTX/GO complexes as compared to free RTX. To test this possibility, Raji cells were incubated with increasing concentrations of unlabeled RTX before being stained with FITC-RTX or FITC-RTX/GO. Pre-incubation with unlabeled RTX diminished staining by FITC-RTX in a dose-dependent manner (Figure 2C). In contrast, the staining intensity by FITC-RTX/GO was not affected by pre-incubation with unlabeled RTX, even when unlabeled RTX was present at substantially higher concentrations than FITC-RTX/GO (3750 μg/ml *vs* 10 μg/ml) (Figure 2C). These results suggest that FITC-RTX/GO has the capacity to replace unlabeled free RTX that are already bound to CD20, whereas free FITC-RTX cannot. Therefore, FITC-RTX/GO appears to have a much higher avidity for CD20 compared to FITC-RTX. Given that avidity is a function of accumulated strength of multiple affinities, the increase in avidity suggests that RTX present in RTX/GO mixtures is multivalent. Immunofluorescence microscopy revealed that free FITC-RTX stained in a uniform manner across cell membranes, but FITC-RTX/GO staining appeared as coarse fluorescent aggregates clustered on the poles of stained cells (Figure 2D), consistent with capping of CD20 [34]. As capping is the result of crosslinking of ligands by multivalent antibodies [35], this result further demonstrates that GO-associated RTX is multivalent. No internalization of the fluorescence was visualized after prolonged incubation of Raji cells with FITC-RTX/GO in culture over a course of 8 hours; at this time, scattered cell aggregates consistent with homotypic aggregation could be identified on a background of single cells.

### GO-associated RTX is directly cytotoxic to malignant B cells

**Figure 3:**
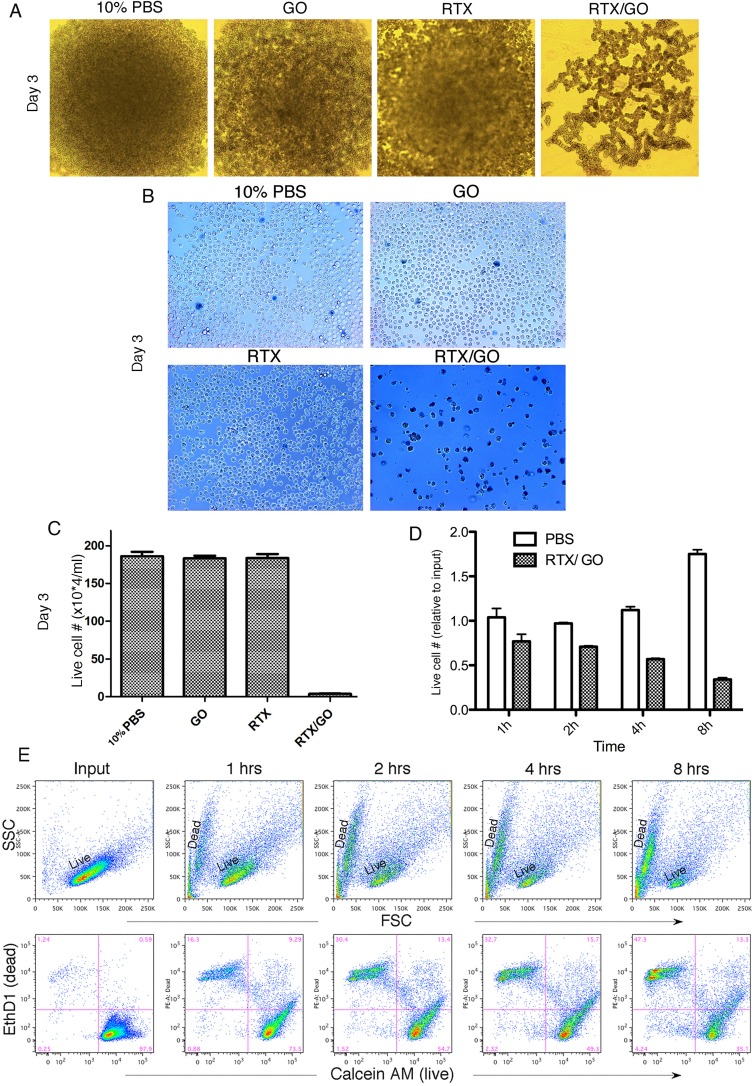
Cytotoxicity of RTX/GO to Raji cells: Microscopic image of undisturbed Raji cells cultured for 3 days with 10% PBS, GO, RTX and RTX/GO shows no appreciable cell proliferation in the culture with RTX/GO **A.** The cultured cells were washed with EDTA-containing buffer and stained with trypan blue to show primarily dead cell debris left in the culture with RTX/GO **B.** Trypan blue-stained Raji cells in the day 3 cultures were enumerated using a hemocytometer, and the number of live cells in each culture was plotted **C.** Raji cells were cultured with RTX/GO or 10% PBS as a control for 1, 2, 4 and 8 hours, and live cells were enumerated using a hemocytometer. The percentage of live cells relative to input cells at each time point was plotted **D.** Flow cytometry shows progressive loss of live cells with accumulation of dead cells as a function of time over the 8-hour culture period as revealed by FSC x SSC display or by the LIVE (Calcein AM) and DEAD (EthD1) dye stain **e.** The results are representative of more than three experiments.

Given previous reports indicating that crosslinking CD20 (CD20XL) causes target cell death [16, 17], the capacity of RTX/GO to crosslink and induce CD20 capping raised the possibility that RTX/GO might be cytotoxic to CD20^+^ cells. To test this, Raji cells were cultured for three days with RTX/GO *versus* 10% PBS, free GO, or free RTX as controls. When the cultures were monitored by naked eye or microscopic examination, robust cell expansion was visualized in all the control cultures but not in the culture with RTX/GO (Figure 3A). When trypan blue staining was performed after washing the cells with EDTA-containing buffer, it was revealed that that the cultures with RTX/GO contained predominantly blue dead cells and debris, with only scattered live cells (Figure 3B). In contrast, nearly all of the Raji cells cultured with 10% PBS, GO, and RTX were viable and unstained by trypan blue. Enumeration of the cells with a hemocytometer confirmed the paucity of live cells in the RTX/GO cultures (Figure 3C). To assess the kinetics of cell death, the number of live cells was enumerated with a hemocytometer at various time points over a period of 8 hours. A small but significant degree of reduction in live cell numbers was detectable by the end of first hour of culture in comparison to the number of input cells, and the reduction progressed steadily as a function of time (Figure 3C). By eight hours, the live cell counts were decreased by approximately 60%, while in the PBS control cultures, Raji cells underwent significant proliferation (Figure 3D). Flow cytometry was also performed in conjunction with LIVE/DEAD cell staining to identify live and dead cells. On FSC x SSC display of flow cytometry, a progressive loss of live cells along with accumulation of dead cell debris was found (Figure 3D). On LIVE/DEAD cell stain, the cell death was reflected by a continuous reduction in the frequency of Calcein AM^+^ live cells and an increase in the frequency of EthD1^+^ dead cell debris (Figure 3E). Thus, the elimination of the target cells by RTX/GO is initiated shortly after its interaction with the cells. These results taken together demonstrate that RTX/GO is cytotoxic with the capacity to rapidly kill Raji cells, while neither RTX or nor GO alone is cytotoxic at the concentrations used, consistent with previous reports [24, 36, 37].

**Figure 4:**
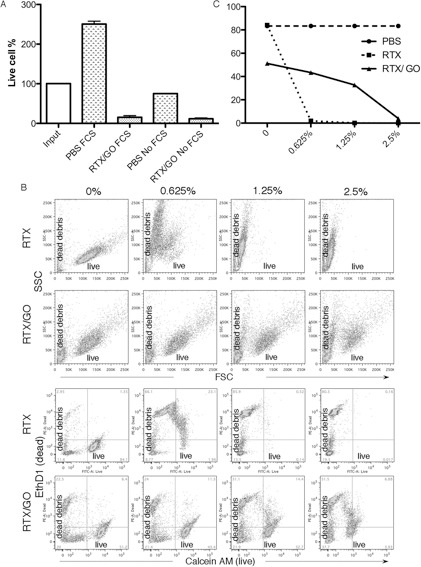
Role of complement in RTX/GO-mediated cytotoxicity: Raji cells were cultured overnight with 10% PBS or RTX/GO in RPMI 1640 media with or without 10% heat-inactivated fetal bovine serum (FBS, Sigma), percentages of live cells relative to the input cells are plotted **A.** Raji cells were opsonized either by RTX (upper panel) or RTX/GO (lower panel) for 2 hours followed by incubation with increasing concentration of rabbit complement for 45 minutes, stained with the LIVE (Calcein AM)/DEAD (EthD1) dye and analyzed by flow cytometry **B.**, and the percentage of live cells is plotted **c.** The results are representative of three experiments.

The fetal calf serum (FCS) in the culture media was heat-inactivated and therefore not expected to contain complement activity. To completely rule out a role of complement in RTX/GO-mediated cytotoxicity, Raji cells were cultured with RTX/GO in serum-free media. Lack of FCS is the culture media affected Raji cell survival and proliferation as compared to the FCS-supplemented media (Figure 4A). However, regardless of the absence or presence of FCS, RTX/GO killed Raji cells to a similar extent under both conditions (Figure 4A). This result confirms that RTX/GO kills Raji cells directly and complement is not required for the cytotoxicity. The anti-CD20 mAbs have been classified into 2 subtypes. Type I rituximab-like anti-CD20 mAbs potently activate complement [38] whereas type II anti-CD20 mAbs weakly activate complement but more potently evoke direct killing [39, 40]. To examine the capacity of RTX/GO to activate complement, complement-dependent cytotoxicity assays was performed as previously described [41]. Raji cells were first cultured with either RTX or RTX/GO for two hours and subsequently exposed to increasing concentrations of rabbit (serum) complement for an additional hour. Flow cytometry in conjunction with LIVE/DEAD cell dye staining was used to analyze cell lysis. As expected, in the absence of complement, RTX/GO but not RTX killed a fraction of Raji cells during the 3-hour culture period (Figure 4B). Adding rabbit complement to RTX-opsonized Raji cells resulted in nearly complete lysis of Raji cells, even at the lowest concentration tested (0.625%) (Figure 4B & 4C), consistent with a previous report of RTX being a potent activator of complement [38]. When a similar range of complement concentrations was added to RTX/GO opsonized Raji cells, this lead to a much smaller degree of Raji cell lysis, as compared to free RTX-opsonized cells (Figure 4B and 4C), indicating that RTX/GO is less potent in complement activation than RTX.

**Figure 5:**
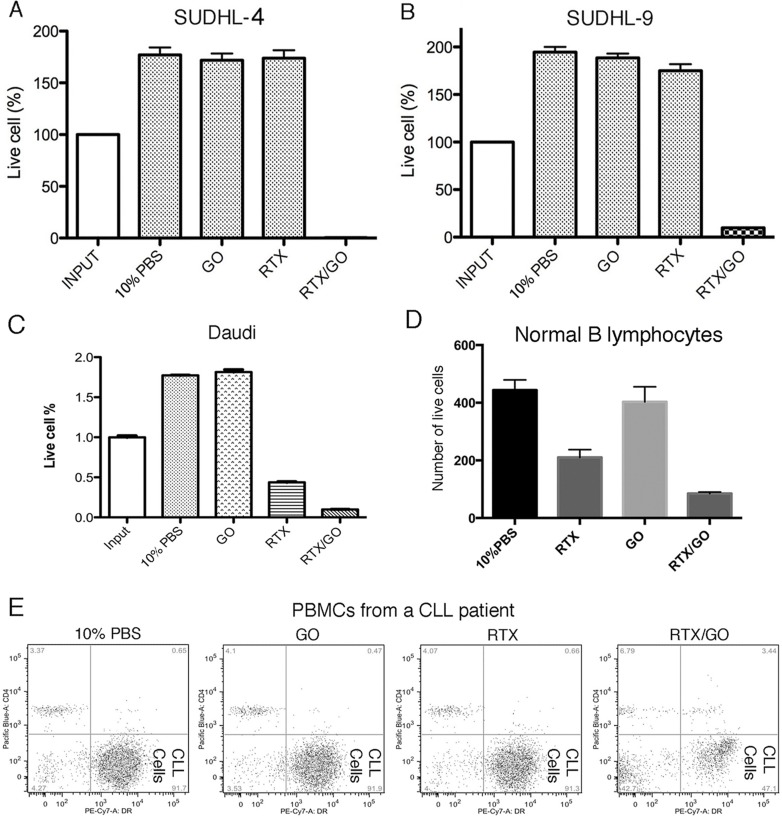
RTX/GO cytotoxicity on other lymphoma cells and normal B-lymphocytes: SUDHL-4 **A.**, SUDHL-8/9 **B.**, Daudi cells **C.**, normal B lymphocytes **D.** and PBMCs from a patient with chronic lymphocytic leukemia **e.** were cultured for one day with10% PBS, GO, RTX or RTX/GO. The bar graph showed the percentages of live cells relative to input cells. The cultures were repeated at least three times.

To examine the potential cytotoxicity of RTX/GO on other types of CD20^+^ lymphoma cells, another Burkitt lymphoma cell line Daudi [42], two diffuse large B cell lymphoma cell lines SUDHL-4 [43] and SUDHL-8/9 [44], primary lymphoma cells from a patient with chronic lymphocytic leukemia, as well as primary B lymphocytes from normal donors were tested in culture as targets of RTX/GO. As shown in Figure 5, RTX/GO directly killed all the tested cells types in the absence of complement. These results suggest the potential of RTX/GO to directly kill a broad range of target cells.

### The actin network is involved in RTX/GO-mediated cytotoxicity

**Figure 6:**
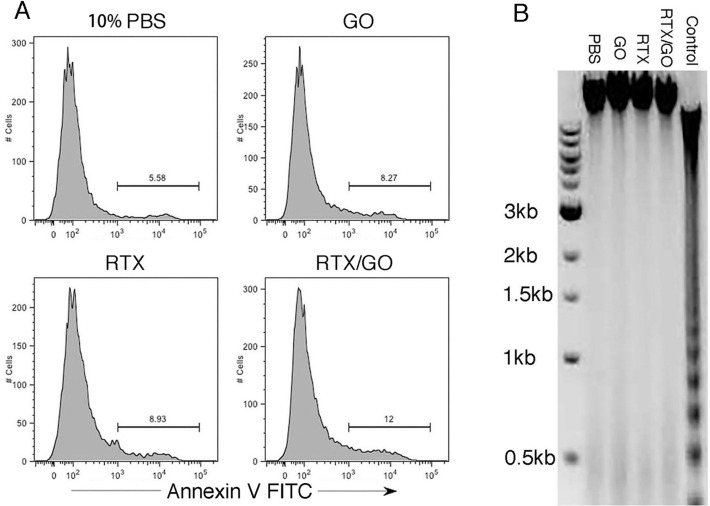
Non-apoptotic nature of RTX/GO-induced cell death: Raji cells were cultured overnight with the indicated treatment, stained with apoptotic marker Annexin V, and analyzed by flow cytometry No gate was applied on the displayed cells **A.** Gel electrophoresis of DNA isolated from Raji cells cultured overnight with 10% PBS, GO, RTX and RTX/GO. DNA isolated from mouse thymocytes that were cultured in plain medium for two days was used as an apoptosis positive control **B.** The experiments were repeated at least three times.

**Figure 7:**
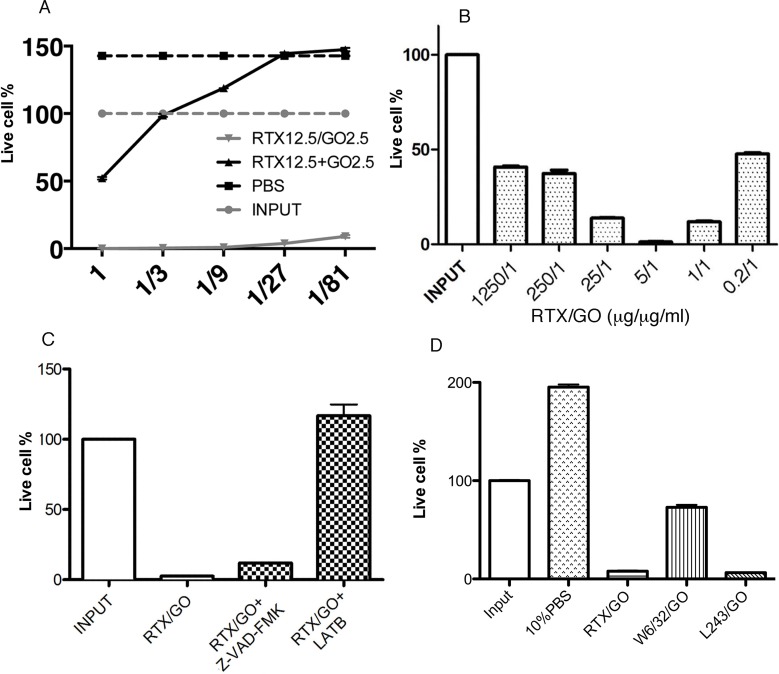
Features of RTX/GO-mediated cytotoxicity: Raji cells were cultured overnight with decreasing concentrations of RTX/GO or RTX plus GO separately added to the culture at the culture initiation The starting concentration of RTX and GO was 12.5 μg/ml and 2.5 μg/ml, respectively **A.** Raji cells were cultured with RTX/GO at the indicated RTX:GO ratio with the concentration of GO fixed at 1 μg/ml. The bar graph showed the percentages of live cells compared to input cells **B.** Raji cells were cultured overnight with RTX/GO, RTX/GO plus Z-VAD-FMK (100 μM) or latrunculin B (LatB) (10 μM) and the percentages of live cells relative to input are plotted in bar graph **C.** Raji cells were cultured with the 10% PBS, RTX/GO, W6/32/GO and L243/GO and the percentages of live cells relative to input are plotted in the graph **D.** The results are representative of multiple experiments.

Previous studies report that CD20-crosslink induces target cell death by apoptosis [16, 31, 39, 45]. On flow cytometry, RTX/GO-killed cells appeared to be necrotic rather than apoptotic, as they rapidly became debris (Figure 3B & 3E), similar to complement-lysed cells (Figure 4B). Annexin V staining did not detect a significant increase in the frequency of Annexin V^+^ cells when compared to the controls (Figure 6A), and DNA electrophoresis did not identify apoptotic DNA fragmentation (Figure 6B), suggesting that cell death was non-apoptotic in nature. In line with these findings, the pan-caspase inhibitor Z-VAD-FMK did not significantly rescue RTX/GO-induced Raji cell death (Figure 7C). Adding unmixed RTX and GO at the initiation of culture did not cause significant cell death, except at very high concentrations of RTX and GO (Figure 7A). This is in sharp contrast to the pre-mixed/incubated mixture of RTX/GO, which killed Raji cells at much lower concentrations (Figure 7A), indicating that physical association between GO and RTX is required for the potent cytotoxicity. When RTX/GO mixtures made with different RTX to GO mass ratios were tested, the highest cytotoxicity was observed at a RTX to GO of 5 to 1, although the mixtures made with other RTX-to-GO ratios were also cytotoxic (Figure 7B). As 5 to 1 mass ratio is the maximum GO binding capacity for RTX as determined in the experiments described above (Figure 1), this result suggests that the highest valence of RTX in RTX/GO gives rise to the strongest cytotoxicity, further supporting the idea that the cytotoxicity of RTX/GO results from an increase in the valence of RTX, which leads to CD20 crosslinking and capping. If CD20 capping is required for RTX/GO-induced cytotoxicity, interruption of this process might alleviate RTX/GO-mediated cytotoxicity. It was previously reported that reorganization of the actin network is required for capping of surface molecules [34]. To determine whether interrupting the actin network reorganization would interrupt RTX/GO-induced cytotoxicity, an actin polymerization inhibitor, latrunculin B (LatB) was used in the Raji cell culture. LatB completely abrogated RTX/GO-induced Raji cell death (Figure 7C), indicating that the actin network is involved in RTX/GO-induced cell death. To determine whether antibodies reactive with other cell surface molecules would similarly be cytotoxic when associated with GO, anti-MHC class I and class II mAb, W6/32 and L243 were used to generate mAb/GO complexes. W6/32 is specific for HLA-A, B and C whereas L243 is specific for HLA-DR. HLA-DR is a lipid raft-associated protein, whereas HLA-A, B and C are located outside of lipid rafts. When Raji cells were cultured with L243/GO or W6/32/GO, cell death was observed in both cultures (Figure 7D), indicating that targeting of other cell surface molecules with mAb/GO may also give rise to cytotoxicity. However, W6/32/GO killed Raji cells at a much lower intensity as compare to L243/GO or RTX/GO (Figure 7D). Since CD20 and HLA-DR are lipid raft-associated proteins but HLA-A, B and C are not [39, 46, 47], these results suggest that the binding of lipid raft-associated proteins by antibody/GO may lead to more extensive cell death as compared to non-lipid raft-associated proteins. Given previous reports that the actin network plays a role in controlling the location and function of lipid raft-associated proteins [48, 49] and that ligation of DR with L243 antibody can induce target cell death in a cytoskeletal function-dependent manner [40, 50], the potent cytotoxicity observed with RTX/GO and L243/GO might be related to the involvement of the actin network. These findings are consistent with the results showing that latrunculin B alleviates RTX/GO-mediated cytotoxicity (Figure 7C).

### RTX/GO but not free RTX rapidly eliminates Burkitt lymphoma in a xenograft lymphoma mouse model

**Figure 8:**
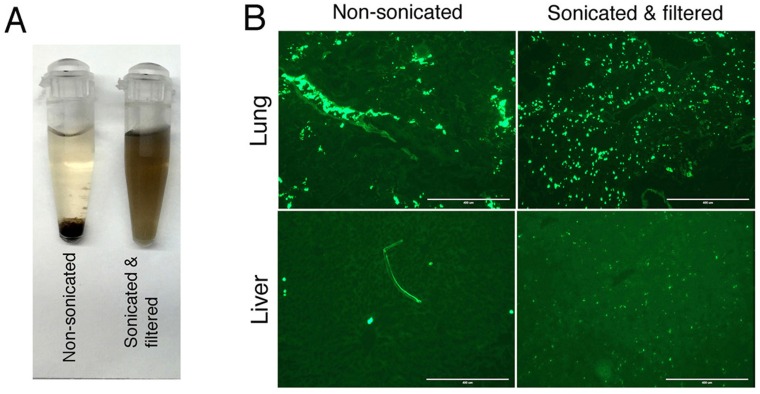
Stability and *in vivo* distribution of FITC-RTX/GO made from non-sonicated or sonicated/filtered GO: Non-sonicated or sonicated/filtered GO was mixed with human serum (10 μg/ml) and the GO serum dispersions were monitored for 21 days Non-sonicated GO precipitated in four hours whereas sonicated/filtered GO remained in dispersion at least by day 21 **A.** RTX/GO made of either non-sonicated or sonicated/filtered GO with RTX-FITC was injected into NRG mice *iv* and organs were sampled 2 hours after injection. Fluorescence microscopy shows the distribution of FITC-RTX/GO in the lung and liver. The images were taken using EVOS Cell Imaging System, (AMF-4301), with scale bar showing 400 μ. The location of fluorescence derived FITC-RTX/GO made of sonicated/filtered GO in the liver is consistent with location of Kupffer cells **B.**

To study therapeutic potential of RTX/GO *in vivo*, high-grade systematic Burkitt lymphoma was established by intravenous *(iv)* injection of Raji cells into immunodeficient NODrag^ko^γ^ko^ (NRG) mice. As NRG mice are deficient in T, B and NK cells along with a defective complement system and macrophage activity [51, 52], they constitute an idea animal model for evaluation of therapeutic capacity of RTX/GO in the absence host effector mechanisms such as CDC and ADCC. Lymphomas that develop in this mouse model primarily involve the liver and bone marrow but not the lymph nodes, spleen, lungs, or other organs, as previously reported [53]. Since the serum half-life and biodistribution of GO *in vivo* may be limited due to large size as reported in previous studies [54], GO used in this study was vigorously sonicated and filtered though a 0.22 μm filter. GO dispersions that were not sonicated were unable to pass through a 0.22 μm filter, but they readily passed through after sonication. Sonicated/filtered GO remained in dispersion in human serum indefinitely while non-sonicated GO precipitated within 4 hours (Figure 8A). After intravenous injection, FITC-RTX/GO made from non-sonicated GO was only visible within the lumens of the lung vasculature but undetectable in the lung tissue or distant organs such as the liver (Figure 8B). In contrast, RTX/GO made with sonicated/filtered GO was readily identifiable in the tissue without intra-vascular retention (Figure 8B).

**Figure 9:**
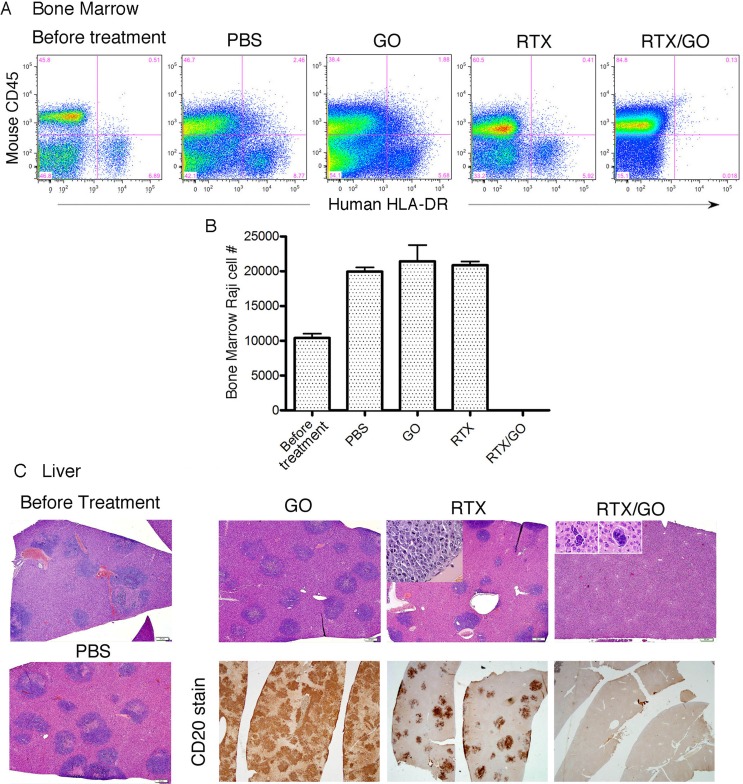
Therapeutic capacity of RTX/GO in NRG mice: Bone marrow cells from lymphoma-transplanted mice before or after receiving the indicated treatments were stained with mouse CD45 and human HLA-DR and analyzed by flow cytometry Raji cells are identified as mouse CD45^neg^ and human HLA-DR^pos^ cells **A.** Raji cell numbers per a given unit volume of bone marrow of the indicated treatment groups are plotted **B.** H&E stained microscopic images (taken with 4x objective lens, scale bars: 50 μ) of the liver show lymphoma (blue) infiltrates present only in the livers of PBS, GO, and RTX-treated mice but not of RTX/GO treated mice **C.** The lack of lymphoma in RTX/GO-treated mouse liver was confirmed by CD20 staining (C, images were taken with 2x objective lens).

Eight days after Raji cell transplantation, extensive lymphoma infiltrates were identified in the bone marrow and liver (Figure 9A-9C); and treatments were then started. Four groups of mice, four to five in each group, were given intravenously PBS, GO, RTX, or RTX/GO respectively every two to 3 days for a total of three treatments. All the mice were sacrificed for analysis 3 days after the last treatment. Extensive human HLA-DR^+^/mouse CD45^−^ lymphoma infiltrates were identified by flow cytometry in the bone marrows of the PBS, GO and RTX-treated animals but not in RTX/GO-treated ones (Figure 9A and 9B), indicating elimination of lymphoma cells at this location by RTX/GO but not by free RTX or GO. The Raji cell counts in the bone marrow of GO- and RTX-treated mice were similar to that of PBS-treated mice (Figure 9B), consistent with the results of the *in vitro* experiments showing that neither GO nor RTX is independently cytotoxic to Raji cells. Histological examination identified infiltrating lymphoma in the livers of all the PBS, GO and RTX-treated mice, which appeared as blue cell infiltrates on H&E stained sections (Figure 9C). Human CD20 staining confirmed that the liver infiltrates were composed of CD20^+^ lymphoma cells (Figure 9C). In RTX-treated mice, lymphoma infiltration was not as dense as in PBS or GO-treated mice, but frequent mitotic figures were readily identified within the lymphoma infiltrates (Figure 9C insert), indicating rapid proliferation and progression of the lymphoma cells despite RTX treatment. In sharp contrast, pathological examination as well as CD20 staining demonstrated no evidence of viable lymphoma in the livers of RTX/GO treated mice (Figure 9C). Only under high magnification microscopy could rare degenerated cellular elements be identified in the liver of some of the RTX/GO-treated mice, consistent with remnants of lymphoma cells killed by RTX/GO. No pathological abnormalities were identified in any major organs examined in the RTX/GO treated mice, including the heart, lung, kidney, liver (except for the presence of remnants of degenerated lymphoma cells), pancreas and GI tract. The physical and functional status of the mice was closely followed during the experiments. No evidence of morbidity of any degree was identified with RTX/GO treated mice, and they remained healthy throughout the course of treatment, suggesting that RTX/GO is non-toxic and safe. Taken together, these results demonstrate that RTX/GO has the capacity to diffuse out of the blood circulation, penetrate through the tissue to reach target cells, and rapidly eliminate established lymphomas in the absence of host effector mechanisms, while free RTX or GO fails to do so.

## DISCUSSION

The findings of the current study represent a novel strategy to substantially improve the therapeutic efficacy of a well-established therapeutic anti-CD20 antibody RTX. There has been continuous investigation towards the development of newer generations of anti-CD20 mAbs aimed at improving therapeutic efficacy over RTX [1, 15, 55]. Various strategies have also been taken to generate multivalent anti-CD20 antibodies with a capacity to crosslink CD20, including utilization of liposomes [56], the Dock-and-Lock (DNL) technology [57], and complementary oligonucleotide-based two-step pre-targeted approaches [32]. Our results demonstrate that GO can serve as excellent scaffold for formation of multivalent antibodies for therapeutic application. Although mediated through non-covalent interaction, the binding between RTX and GO is stable (Figure 1). The multivalent nature of RTX in the form of RTX/GO is reflected by the substantial increase in the antibody avidity and the capacity of RTX/GO to crosslink and cap CD20 (Figure 2).

Extensive CD20 crosslinking by multivalent anti-CD20 antibodies tends to intensify various cellular signals, including the induction of apoptosis and inhibition of cell growth/survival. While the molecular mechanism of RTX/GO-mediated cytotoxicity remains to be delineated, our results are consistent with previous reports showing that crosslinking CD20 (CD20XL) causes target cell death *in vitro* [16, 45]. It has been reported in recent studies that GO at concentrations greater than 20 or 50 μg/ml may cause cell death by oxidative stress [21, 24]. In our experiments, the concentration of GO in RTX/GO used to effectively kill Raji was at least as low as 0.2 μg/ml (Figure 5C), suggesting that RTX/GO might mediate cytotoxicity through mechanisms other than oxidative stress. The observed abrogation of cell death by the actin inhibitor LatB suggests that RTX/GO induces cell death through an actin dependent process. Nonetheless, the data presented in the current study does not exclude the possibility that oxidative stress may contribute to the RTX/GO-mediated cytotoxicity, as GO can be concentrated onto the target cells through RTX/GO binding to CD20. While CD20XL-induced cell death has been reported to be apoptotic in previous studies [16, 17], our results suggest that RTX/GO kills the target cells though a non-apoptotic mechanism (Figures 5 & 7). A non-apoptotic mechanism of RTX/GO-mediated killing might provide therapeutic advantage because faulty apoptotic pathways can contribute to mechanisms of cancer therapy resistance [43, 58, 59]. Studies have shown that when GO is absorbed to lipid membranes, it can cause liposomes to rupture [28]. It is possible that GO becomes absorbed into the plasma membrane of target cells as a result of RTX/GO-mediated CD20 capping, which leads to plasma membrane damage and rapid cell death. Since capping is an actin-dependent process, LatB might prevent cell death by interrupting CD20-cap formation. Alternatively, LatB might prevent cell death by inhibiting actin network disorganization induced by CD20 capping. Studies have reported rapid cell death resulting from damage to actin networks [60].

The serum half-life of RTX/GO may be short as compared to that of free RTX, according to previous reports [61]. However, an extended serum half-life may not be necessary for the RTX/GO therapeutic effect, as RTX/GO appears to eliminate lymphoma rapidly (Figure 8). The capacity of RTX/GO to directly kill lymphoma cells in the absence of the host effector mechanisms may have important therapeutic implications. RTX/GO therapy might be effective even in patients whose host effector mechanisms are compromised as discussed above [5-7]. It is believed that refractory disease and relapse with NHL may result from acquired resistance to RTX, which occurs as a result of downregulation of CD20 expression [62]. One of the primary mechanisms for CD20 downregulation is stripping or shaving of the molecule from the cell surface by macrophages, and this may occur as a result of binding of RTX to CD20 without subsequent elimination of the cells in the absence or exhaustion of the host effector mechanisms such as CDC and ADCC [6-9]. The capacity of RTX/GO to directly kill lymphoma cells may negate this resistance mechanism.

While further studies are needed to determine the impact of RTX/GO therapy on long-term survival, the current data suggest the possibility that RTX/GO might be significantly more effective than free RTX. Indeed, despite the potent capacity to activate complement and ADCC, RTX in general is not very effective when used as monotherapy in the induction therapy of high grade lymphomas, suggesting limited efficacy of the host effector mechanisms in eliminating lymphoma cells. The capacity of RTX/GO to eliminate lymphoma even in the absence of host effector mechanism suggests the possibility that this formulation might be effective for treatment of NHL in patients even when used as a monotherapy. If effective as monotherapy, RTX/GO treatment could make chemotherapy unnecessary. Omitting chemotherapy would not only avoid serious toxic complications, but also preserve the function of immune system, providing an optimal context for immunotherapy that might significantly improve the prognosis of NHL patients [63-66].

## MATERIALS AND METHODS

### Cell culture

Raji, Daudi, SU-DHL4, and SU-DHL8/9 cell lines were originally obtained from ATCC (Manassas, VA, USA). PBMCs were obtained from normal donors or a patient with CLL in accordance with the institution-approved IRB. The cells were cultured in RPMI 1640 media supplemented with 10% heat-inactivated fetal bovine serum (FBS, Sigma), 1× Penicillin-Streptomycin-Glutamine (PSG), 1×2- Mercaptoethanol, 1× sodium pyruvate, and MEM Non-Essential Amino Acids (NEAA), at 37°C in a humidified atmosphere of 5% CO_2_. All cell culture components were obtained from Life Technologies.

### Loading RTX onto graphene oxide

GO dispersions were purchased from Sigma. To reduce the size, GO dispersions were sonicated with a probe sonic dismembrator (Fisher Scientific; Model 550) for 120 minutes at amplitude of 3.5, followed by filtration through a 0.22μm filter. To load RTX onto GO, 0.2 mg GO and 1 mg RTX were mixed in 1 ml 10% PBS (0.09% NaCl), undiluted PBS (0.9% NaCl), or water according to the experiments, and incubated at 37°C for 12 hours under constant agitation. The RTX/GO mixtures were used for staining, cell culture, and *in vivo* experiments. To examine binding between RTX and GO and determine the amount of RTX bound to GO, GO was precipitated from RTX/GO mixtures and was washed and boiled in SDS gel-loading buffer with dithiothreitol (DTT), and subjected to 12% SDS polyacrylamide gel electrophoresis (PAGE).

### FITC conjugation of RTX and staining

RTX (Roche) was obtained from the University of Utah Hospital Pharmacy. Two milligrams of Fluorescein-5-isothiocyanate (FITC) (Life Technologies) were dissolved in 200 μl anhydrous DMSO (Fisher) immediately before use. Dissolved FITC (40 μg) was added to 1 mg RTX in 1 ml. After incubation in darkness on a rotating platform for 1 hour at room temperature, the reaction was dialyzed in a cold room in PBS for 48 hours with 3 changes of PBS. FITC-conjugated RTX was loaded onto graphene oxide similarly to the loading of RTX as described above. To stain Raji cells, FITC-RTX or FITC-RTX/GO was added to Raji cell cultures at 10 μg/ml and mixed every hour. Aliquots of cells were taken out of culture at different culture time points, washed and fixed for flow cytometry. A proportion of fixed cells was mounted on a glass slide, and sealed under a cover slip with a gold antifade reagent with DAPI (4′ 6-diamidino-2- phenylindole) (Invitrogen). Images were taken using an Olympus FV 1000 confocal microscope at the Fluorescence Microscopy Core Facility, a part of the Health Sciences Cores at the University of Utah. Microscopy equipment was obtained using a NCRR Shared Equipment Grant # 1S10RR024761-01. The LIVE/DEAD^®^ Viability/Cytotoxicity Assay Kit containing calcein AM and ethidium homodimer (EthD-1) from Life Technologies was used to identify the live and dead cells according to the vendor provided protocol, and the stained cells were analyzed within 1 hour by flow cytometry using a LSR Fortessa (BD Biosciences, San Jose, CA). Annexin V/PI staining kits were obtained from BD Biosciences. Cells were washed with cold PBS before being stained with Annexin V (1:10 binding buffer) and 20 μl PI (1:10 binding buffer). After incubating in the darkness for 15 minutes, 200 μl binding buffer was added to each sample according the vendor's protocol. Flow cytometry was performed within one hour.

### Complement dependent cytotoxicity assay

Raji cells were cultured at 37°C with 10%PBS, RTX and RTX/GO for 2h and then incubated increase concentration (0.625%, 1.25%, 2.5%) of rabbit complement (Abdserotec, USA) for 45 minutes. The cells were then stained with LIVE/DEAD cell dye and analyzed by flow cytometry.

### Xenograft human Burkitt lymphoma mouse model

The immunodeficient NOD-rag-/—γ−/− mice were purchased from Jackson Laboratory, USA, and housed at the University of Utah animal facility according to standard guidelines. To establish systemic lymphoma, 4 × 10^6^ Raji cells were injected into six to 10 weeks old mice *via* the dorsal tail vein. Male and female mice were randomly included in experiments. Eight days after Raji cell transplantation, the mice were treated with *iv* injection of PBS, GO, RTX or RTX/GO at 1 μg GO/g and 5 μg RTX/g of mouse body weight on days 8, 9, 10, and 12. Mice were sacrificed on day 12-14 for pathological examination and flow cytometric analysis. Major organs of were harvested and fixed in 10% formalin, embedded in paraffin, sectioned, and stained with hematoxylin and eosin (H&E staining) or anti-CD20. Histological examination was conducted by a Board-certified pathologist. The study was carried out in strict accordance with the recommendations in the Guide for the Care and Use of Laboratory Animals of the National Institutes of Health under a protocol provided by the Institutional Animal Care and Use Committee (IACUC) at the University of Utah (Protocol 14-0513).

## Abbreviations

GO: graphene oxide
RTX: rituximab
RTX/GO: a pre-incubated mixture of RTX and GO
ADCC: antibody-dependent cell-mediated cytotoxicity
CMC: complement-mediated cytotoxicity
